# A two year national surveillance for *Aethina tumida* reflects its absence in Spain

**DOI:** 10.1186/1756-0500-7-878

**Published:** 2014-12-05

**Authors:** Almudena Cepero, Mariano Higes, Amparo Martínez-Salvador, Aránzazu Meana, Raquel Martín-Hernández

**Affiliations:** Centro Apícola de Marchamalo, Consejería de Agricultura, Castilla-La Mancha, Camino de San Martín s/n, 19180 Guadalajara, Spain; Epidemiology Consultant, C/Puente la Reina, 28050 Madrid, Spain; Animal Health, Facultad de Veterinaria, Universidad Complutense de Madrid, Avda. Puerta de Hierro s/n, 28040 Madrid, Spain; Fundación Parque Científico y Tecnológico de Albacete (INCRECYT- FEDER), Madrid, Spain

**Keywords:** *Apis mellifera*, Small hive beetle, *Aethina tumida*, Surveillance

## Abstract

**Background:**

The Small Hive Beetle (SHB) is considered one of the major threats to the long-term sustainability and economic success of honey bee colonies in Europe. The risk of introduction into the EU had been reported as moderate to high. Indeed, it has been recently reported an outbreak in the south of Italy. Here, the presence of *Aethina tumida* in beekeeping farms in Spain was evaluated using a previously described qPCR protocol.

**Findings:**

When hive debris from 398 colonies (collected in 2010 and 2011) was analysed, grouped by region, SHB were not detected in any of the samples, making it unnecessary to analyse the samples individually.

**Conclusion:**

The SHB free-status is shown. This epidemiological surveillance would appear to be useful to detect the possible future entry of this pathogen.

## Findings

### Introduction

Exotic diseases or pests are infectious, parasitic diseases or arthropods that are not native to a particular region either because they have never been present there or because they were eradicated and then kept out by government control measures. The impact of exotic agents varies considerably depending on the species and the area under invasion. International and government bodies enforce specific regulations and policies to avoid the entrance and/or dissemination of such exotic organisms, establishing preventative controls and contingency plans.

In terms of beekeeping, the Small Hive Beetle (SHB; *Aethina tumida*) is considered one of the major worldwide threats to the long-term sustainability and economic success of colonies in Europe [1]. This beetle is originated from Africa where it is considered a minor pest of bee colonies, causing little harm, however the beetle can multiply to huge numbers within infested colonies in other geographical areas. Since SHB was discovered outside or its native range in 1996, it has been reported in many different countries including the United States, Canada, Australia, Mexico, Jamaica and Cuba where it has spread [1–4]. The larvae of this beetle eat brood, honey and pollen; they destroy combs and cause fermentation, spoiling the honey. These severe consequences of infestation become further exaggerated as the beetle populations become established in these areas with little or no chance of eradication [3] although some biological requirements of the beetles may limit/enhance their reproductive potential in various soil environments (especially in dry climates) [5].

In 2004, the alarm of infestation was raised in Portugal, although the affected colonies were destroyed immediately [4]. Very recently, Italy has reported an outbreak in the south of the country. It started in Reggio di Calabria but nowadays SHB has spread up to the near province of Vibo Valentia. Up to 4 November 2014, adults of *A. tumida* have been observed in 52 apiaries [6, 7]. Previously, the Panel on Animal Health and Welfare of the European Food Safety Authority (EFSA) [8] had reported a moderate to high risk of the introduction of SHB into the EU through the importation of live queen bees, swarms and colonies from third countries, and due to the trafficking of other bee products and of accidental bee importation. Indeed, non-bee products as ripe fruits, used beekeeping equipment and soil also represent a moderate risk for SHB entry.

Surveillance and reporting are important practices in the early detection of exotic pests and diseases, helping to maintain effective biosecurity controls. In beekeeping, these activities involve collecting, analysing and interpreting information on the presence or absence of pests and diseases, and the reporting of any unusual or suspect detection to the relevant authorities. In preparation for the detection of exotic pests and the supervision of other common honey bee diseases, a surveillance program has been developed over recent years in Spain. As part of this study, the possible presence of *A. tumida* in honey bee colonies in Spain is evaluated by qPCR, employing a protocol previously described to detect this pathogen in hive debris [9].

## Material and methods

**Figure 1:**
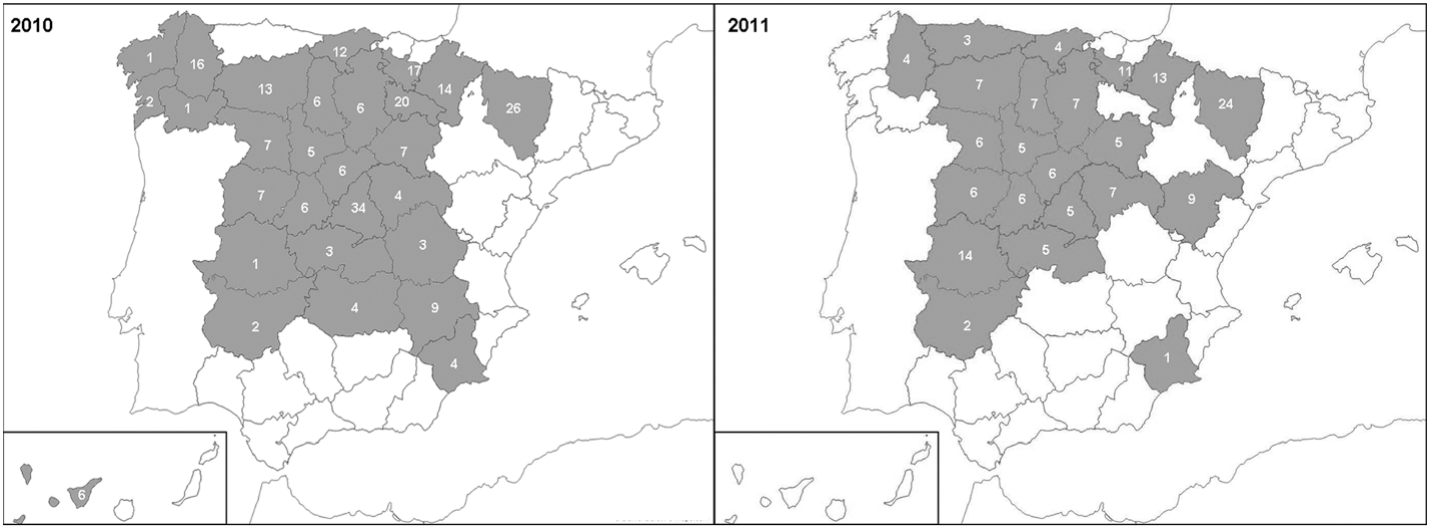
**Distribution of the hive debris samples received in 2010 and 2011.** Samples *were grouped a*ccording to the region of origin for analysis.

The monitoring study was carried out during 2010 and 2011. This program involved active and passive samplings and it was designed to determine the prevalence of most honey bee pathogens and of pesticides around the country. Consequently, the number of colonies to be sampled was calculated according to the number of apiaries registered in 2009 [10], with an expected prevalence of the main honey bee pathogens of around 40%, a precision rate of 10% and a confidence level of 95% as developed in a previous study [11]. Samples were then studied in accordance with the number of apiaries in each region, from which colonies were selected at random (Figure 1).

Each participating beekeeper or the veterinary services of each region were asked to send a plastic jar (125 ml) with the debris of the bottom of six hives randomly selected (bees and brood from the same six hives were also sent for other different studies). As such, a total of 398 samples of hive debris (8.5 g per the six colonies as average) were collected (241 in 2010 and 157 in 2011) and transferred to the laboratory. Given that *A. tumida* had not previously been detected in Spain, the hive debris samples were pooled by regions (Figure 1) for an initial analysis, after which single sample analysis could be carried out in the case of detection.

To prepare the grouped samples, 5 g from each sample of debris was introduced into a double bag strainer (Seward, BA6040) and after the addition of 18 ml of milliQ water (MQW), the sample was crushed in a Stomacher 80 (Biomaster) for 120 seconds. Subsequently, 9 ml of MQW was added and the suspension was homogenised again (60 secs). The macerate obtained was collected in a 50 ml tube and centrifuged at 1,751 x *g* for 10 minutes, the supernatant was discarded and 1 ml of MQW was added to the pellet. Afterwards, an aliquot of 50 mg (on average) from the pellet corresponding to a region was re-suspended in 3 ml of MQW and homogenised, and 400 μl were added to a 96-well plate and processed for DNA extraction as described [12], some wells with water alone were included as negative controls. The plates were then frozen at −20°C until use. Positive controls of SHB larvae and adults (kindly provided by Dr J. Ellis) were included too.To assess whether the system was suitable to detect the SHB when present at a low levels, one SHB larvae was added to one sample of hive debris and processed as described above. This sample was grouped with the samples from the region of Madrid collected in 2010, which represented the most unfavourable situation, where 34 samples were grouped for the collective analysis (Figure 1), and this sample acted as a second positive control.

Real-time PCR was performed in a LightCycler 480 (Roche) using the LightCycler 480 Probes Master mix (Roche, 04887301001), and PCR were carried out as described by Ward et al. [9]. The efficiency of the PCR reaction was assessed using DNA extracted from 2 SHB larvae and 2 adult SHB per triplicate.

## Results

We analysed samples coming from 10 Autonomous Communities. The analysed regions represented the 59.62% in 2010 and 57.13% in 2012 of the total beekeeping farms declared in Spain for those years [10].

As expected, all the hive debris samples analysed from Spanish colonies were negative and thus, further analysis of the independent samples was not necessary. The method used was able to detect the DNA from just one SHB larva in the second positive control sample, validating the failure to detect the pathogen in the samples. The efficiency of the PCR that we get was 93.35% for SHB larvae and 95.5% for SHB adults. Accordingly, the method described proved to be a valuable tool in the surveillance efforts to identify the appearance of this species, since it permits rapid screening of hive debris, as it can detect DNA from SHB eggs, larvae and adult specimens [9].

## Discussion

Effective control of honey bee pests and diseases is important to maintain sustainable and healthy honey bee populations for pollination and honey production, thereby contributing to national and international food security and biodiversity objectives. Although SHB had not been reported or suspected previously in our country, the only way to guarantee its absence is through epidemiological surveys that validate the SHB pest free status. Moreover, Spain is supposed to be a susceptible area for SHB pest establishment since its share climatological and biogeographical characteristics with other countries, where the beetle has been able to spread out. Indeed, the most of Spain presents ecological zones similar than those at the Southern Italy where the SHB has been reported in September, 2014.

The fact that SHB have managed to establish populations in many territories of the USA and Australia suggested that late recognition of this pest in an area prevents its eradication [1] and that supports the necessity of the development of surveillance systems implemented in every country. For example, the identification of this pest was reported two years after its introduction in USA [1, 13] and 12–18 months after the first alert by beekeepers suspicion in Australia (cited in [1]).

Despite strict controls at customs, uncontrolled trading may lead to the invasion of exotic pests or diseases within a territory. Our system of epidemiological surveillance could be useful to detect the possible entry of this pathogen in our country at an early stage of invasion. Even though the surveillance method was design in a broader study to be able to detect other pathogens, not only SHB, the precision rate of this study have a good reliability for the detection of this pest as we have analysed the hive debris from 6 colonies per apiary distributed in areas with a high census of colonies. All the analysed samples were negative, confirming that Spain was free of this pest during the sampled years. Although the veterinary services were asked to be directly involved in the collection of the samples, finally some beekeepers sampled by themselves and this could bias the randomly selection somehow. In any case, there is a very low probability that this could modify the results on the SHB free status of the country.

This monitoring system allows a large number of samples to be processed rapidly, and in an economical and reliable way. Actions such as that described are important for protecting beekeeping activity by detecting exotic pests and diseases early, and to validate control and regulatory measures for established pests and diseases. After the recent outbreak in Italy, measures for monitoring and control for the eradication of the parasite include inspections in all apiaries within a radius of 20 km from the place of the event locations were transhumance is practicing [6, 7]. The implantation of a surveillance procedure in each country together with the setting of the molecular technique [9] for a fast and accurate detection could help to limit the spreading of this pest in areas monitored around outbreaks.
